# Retraction Note: Chemerin reverses neurological impairments and ameliorates neuronal apoptosis through ChemR23/CAMKK2/AMPK pathway in neonatal hypoxic–ischemic encephalopathy

**DOI:** 10.1038/s41419-025-08183-x

**Published:** 2025-10-28

**Authors:** Yixin Zhang, Ningbo Xu, Yan Ding, Desislava Met Doycheva, Yiting Zhang, Qian Li, Jerry Flores, Mina Haghighiabyaneh, Jiping Tang, John H. Zhang

**Affiliations:** 1 Department of Neurology, The First Affiliated Hospital of Chongqing Medical University, Chongqing, 400016 China; 2 Department of Physiology and Pharmacology, Basic Sciences, School of Medicine, Loma Linda University, Loma Linda, CA 92354 USA; 3 Departments of Anesthesiology, Neurosurgery and Neurology, Loma Linda University School of Medicine, Loma Linda, CA 92354 USA

Retraction Note to: *Cell Death* *and* *Disease* 10.1038/s41419-019-1374-y, published online 04 February 2019

The Editors-in-Chief have retracted this article. After publication, concerns were raised regarding highly similar images within the article and with another publication from the same Authors. Specifically:

- Fig. 2A, 9ug/kg and Fig. 6A, Rh-chemerin appear to overlap (resized)
- Fig. 3A, ChemR23-siRNa and Fig. 5A, r-Robo1 [1] appear to be highly similar
- Fig. 3A, Vehicle and Fig. 3A, ChemR23 siRNA appear to partially overlap
- Fig. 3A, Sham and Fig. 3A, scramble siRNA appear to partially overlap
- Fig. 5A, Rh-Chemerin (second and third brain slice from the top) and Fig. 4A, r-Slit2 (second and third brain slice from the top) [1] appear to be highly similar

The Authors confirmed only some similarities but were unable to provide a sufficient response to the other concerns raised. Therefore, the Editors-in-Chief no longer have confidence in the presented data.

None of the authors responded to correspondence from the Publisher about this retraction notice.
